# Impact of Obesity on Outcomes after Minimally Invasive Mitral Valve Surgery: A Systematic Review and Meta-Analysis

**DOI:** 10.1016/j.ijcha.2026.101944

**Published:** 2026-05-22

**Authors:** Paul C. Onyeji, Sonise Momplaisir-Onyeji, Abhigyan Majumdar, Przemysław Nowakowski, Felipe S. Passos, Hristo Kirov, Ricardo Esper Treml, Torsten Doenst, Sophie Tkebuchava, Tulio Caldonazo

**Affiliations:** a All Saints University School of Medicine, Roseau, Dominica; b American University of Barbados, School of Medicine, Bridgetown, Barbados; c GMERS Medical College and Hospital, Sola, Ahmedabad, India; d Medical University of Silesia, Zabrze, Poland; e Department of Thoracic Surgery, MaterDei Hospital, Salvador, Brazil; f Department of Cardiothoracic Surgery, Jena University Hospital, Jena, Germany; g Department of Anesthesiology, Perioperative and Pain Medicine, Stanford University School of Medicine, United States

**Keywords:** Body Mass Index, Minimally Invasive Surgery, Mitral Valve

## Abstract

**Background**: Minimally invasive mitral valve surgery (MIMVS) is increasingly used as an alternative to conventional sternotomy for patients with mitral valve pathologies. This systematic review and meta-analysis aimed to evaluate whether obesity (BMI ≥30 kg/m^2^) is associated with different perioperative and postoperative outcomes in patients undergoing MIMVS.

**Methods**: MEDLINE, EMBASE, and Cochrane Library were systematically searched to identify studies comparing outcomes between BMI groups in patients undergoing MIMVS. The primary outcome was in-hospital mortality. The secondary outcomes were stroke, re-exploration for bleeding, intensive care unit (ICU) length of stay (LOS), hospital LOS, postoperative atrial fibrillation (POAF), requirement of permanent pacemaker implantation (PPI), duration of mechanical ventilation, and wound complications. A random-effects model was performed.

Results

Six studies comprising 5,925 patients met the inclusion criteria. Compared with BMI <30 kg/m^2^, obesity was not associated with higher in-hospital mortality (RR 1.29; 95%CI 0.67 to 2.48; p=0.446; I^2^=31.6%). However, obese patients had significantly higher rates of postoperative atrial fibrillation (RR 1.28; 95%CI 1.12 to 1.46; p<0.001; I^2^=0%), longer intubation duration (MD 0.88; 95%CI 0.11 to 1.65; p=0.02; I^2^=35.7%), longer hospital LOS (MD 0.51; 95%CI 0.04 to 0.99; p=0.03; I^2^=82.3%), and higher requirement for permanent pacemaker implantation (RR 1.43; 95%CI 1.01 to 2.01; p=0.043; I^2^=0%). No significant differences were observed in stroke, ICU LOS, re-exploration for bleeding, or wound complications. Sensitivity analyses confirmed the robustness of the pooled estimates.

Conclusions

In patients undergoing MIMVS, obesity was associated with higher rates of POAF and modestly prolonged postoperative recovery, without increased in-hospital mortality.

## 1 INTRODUCTION

Obesity is a complex, multifactorial chronic disease that substantially contributes to global morbidity and mortality. Its prevalence has risen dramatically over recent decades, with global rates tripling and approximately 40% of men now classified as overweight (body mass index, BMI: 25–30 kg/m^2^) and 13% as obese (BMI > 30 kg/m^2^) [1], [2]. Nevertheless, elevated BMI is consistently associated with adverse health outcomes, with an estimated 67.5% of BMI‑related deaths attributable to cardiovascular disease [2], [3].

In cardiovascular surgery, the impact of obesity on perioperative risk continues to be debated. Some analyses suggest an “obesity paradox,” reporting lower postoperative mortality among obese patients undergoing cardiac surgery, even after attempts to mitigate residual confounding and reverse causation [3], [4]. In contrast, other studies demonstrate that obesity confers substantial procedural risk, including nearly a five-fold increase in deep sternal wound infection and a 25% higher likelihood of requiring renal replacement therapy [3], [4]. Minimally invasive mitral valve surgery (MIMVS), performed via mini-thoracotomy, robotic-assisted, or video-assisted approaches, has been associated with shorter hospital length of stay, reduced blood product utilization, and lower rates of deep wound infection compared with conventional sternotomy, although no consistent survival advantage has been demonstrated in randomized evidence [5]. However, obesity may introduce additional technical challenges in this setting, including increased chest wall thickness, reduced instrument maneuverability, more difficult femoral cannulation, and impaired respiratory mechanics during single-lung ventilation [1], [6], [7], [8]. These factors have historically led some surgical teams to consider elevated BMI a relative contraindication to MIMVS [7], [8].

Despite growing recognition of these technical challenges, the clinical impact of obesity on perioperative outcomes following MIMVS remains uncertain, as the available evidence is largely limited to small single-center observational studies. Therefore, this meta-analysis aims to systematically evaluate the influence of BMI on perioperative outcomes following MIMVS by synthesizing the available evidence comparing patients with BMI ≥30 kg/m^2^ versus BMI <30 kg/m^2^, with the objective of determining whether obesity modifies procedural risk, affects postoperative recovery, or impacts overall clinical outcomes in patients undergoing MIMVS.

## 2 METHODS

This systematic review and meta-analysis was conducted in accordance with the Preferred Reporting Items for Systematic Reviews and Meta-Analyses (PRISMA) guidelines [9], [10]. The review was registered in the International Prospective Register of Systematic Reviews (PROSPERO ID: CRD420261287211).

**Search strategy**

A comprehensive literature search was performed on PubMed, EMBASE, and Cochrane library from inception to April 2026. The search strategy combined controlled vocabulary (MeSH and Emtree terms) and free-text keywords related to obesity and minimally invasive mitral valve surgery. The following search structure was applied: (“mitral valve surgery” OR “mitral valve repair” OR “mitral valve replacement”) AND (“minimally invasive” OR “mini-thoracotomy” OR “robotic” OR “video-assisted”) AND (“obesity” OR “body mass index” OR BMI OR obese). The detailed search strategy is presented in Supplementary Table 1.

**Study Selection**

Two independent reviewers (PO and SM) screened the records, after de-duplication. Any discrepancies and disagreements were resolved by a third and fourth author (FP and TC). Titles and abstracts were reviewed against pre-defined inclusion and exclusion criteria. The reference lists of all included studies were also manually screened to identify additional potentially eligible articles not captured by the primary database search. Study selection and data management were performed using a standardized Microsoft Excel spreadsheet.

## 3 Certainty of Evidence

The overall certainty of evidence for each outcome was assessed independently by two authors (PO and AM) using the Grading of Recommendations Assessment, Development, and Evaluation (GRADE) framework [11]. Because all included studies were non-randomized observational investigations, evidence was classified as low certainty by default. Certainty ratings were subsequently downgraded for: (i) risk of bias (serious or moderate ROBINS-I ratings); (ii) inconsistency (substantial or considerable heterogeneity, I^2^ ≥50%); and (iii) imprecision (wide confidence intervals crossing the null, or small total event counts). No upgrading criteria were applied, as no outcome demonstrated a sufficiently large effect size or a clear dose-response relationship.

**Eligibility Criteria**

Inclusion in this meta-analysis was restricted to studies that met all the following eligibility criteria: 1) randomized controlled trials (RCTs) or observational studies; 2) published in English; 3) reporting comparative outcomes between patients with BMI <30 kg/m^2^ versus BMI ≥30 kg/m^2^ undergoing MIMVS; and 4) reporting at least one outcome of interest. Exclusion criteria included studies involving animal models, case reports, conference abstracts; and no comparative study designs.

**Quality assessment**

Two authors (AM and PM) independently assessed the quality of included studies using the Cochrane Collaboration tool for assessing the risk of bias in non-randomized studies (ROBINS-I) [12]. Disagreements were resolved by consensus.

**Data extraction and baseline characteristics**

Two authors (PO and SM) independently performed data extraction. Disagreements were resolved by consensus. The extracted variables included study characteristics (author, year of publication, country, sample size, and reported outcomes), as well as patient characteristics including age, hypertension, diabetes, chronic obstructive pulmonary disease, and New York Heart Association (NYHA) functional class.

**Outcomes**

The primary outcome was in-hospital mortality. The secondary outcomes were stroke, re-exploration for bleeding, intensive care unit (ICU) length of stay (LOS), hospital LOS, postoperative atrial fibrillation (POAF), requirement of permanent pacemaker implantation (PPI), duration of mechanical ventilation, and wound complications.

### 3.1 Statistical analysis

Statistical analyses were restricted to outcomes reported in at least three studies. For binary outcomes, risk ratios (RR) with 95% confidence intervals (CI) were calculated. For continuous outcomes, mean differences (MD) or standardized mean differences (SMD) with corresponding 95% CI were computed, depending on whether studies used uniform measurement scales. When necessary, means and standard deviations were derived from medians, interquartile ranges, or other summary statistics according to the Cochrane Handbook for Systematic Reviews of Interventions.

Heterogeneity across studies was assessed using the Cochran Q test and the I^2^ statistic. Values of approximately 30%, 50%, and 75% were interpreted as representing moderate, substantial, and considerable heterogeneity, respectively. A p-value <0.05 was considered statistically significant. DerSimonian–Laird random effects models were used for all endpoints. To assess the robustness of the findings, leave-one-out sensitivity analysis was performed for the main outcome and for the outcomes with significant heterogeneity. The Cochrane Handbook for Systematic Reviews of Interventions was used for data handling and conversion. Given the limited number of studies included, meta-regression analyses were not feasible. All statistical analyses were performed using R (version 4.4.0, R Foundation for Statistical Computing, Vienna, Austria).

## 4 RESULTS

**Study characteristics**

**Fig. 1:**
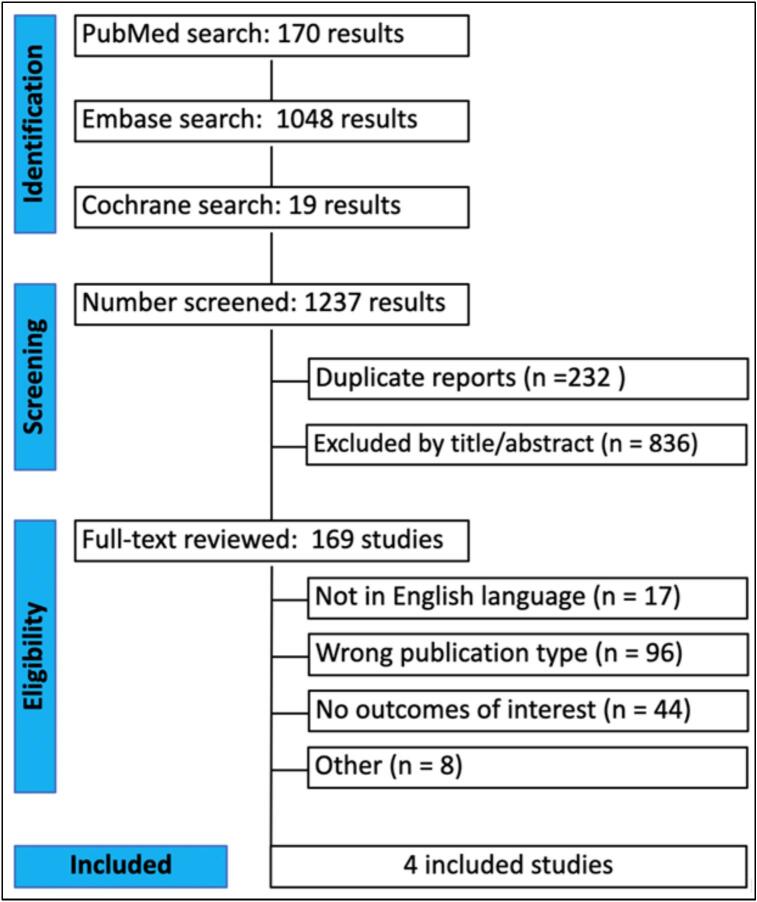
Preferred Reporting Items for Systematic Reviews and Meta-Analyses (PRISMA) flow diagram.

Fig. 1 shows the PRISMA flow diagram outlining the study selection process. The search strategy identified 1,237 results. After deduplication and exclusion based on title and abstract, 169 studies remained for full-text review. Of these, six studies met all the inclusion criteria for the analysis [1], [6], [7], [13], [14], [15].

**Quality assessment and Publication bias**

**Fig. 2:**
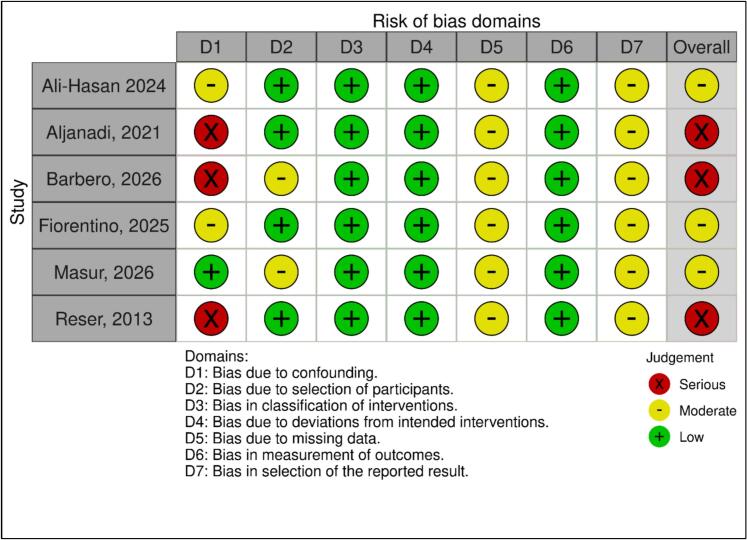
Risk of bias assessment.

The quality of the included studies was assessed using the ROBINS-I tool. Three studies [6], [7], [15] were judged to be at serious risk of bias, primarily due to confounding inherent to retrospective observational designs and baseline imbalances between groups. The remaining three studies [1], [13], [14] were considered to have a moderate risk of bias. Across studies, confounding represented the main source of potential bias, whereas the risk related to intervention classification, deviations from intended interventions, and outcome measurement was consistently low. The overall methodological quality of the included studies is summarized in Fig. 2. Publication bias was not formally assessed because fewer than ten studies were available per outcome.

**Patient characteristics**

The baseline characteristics of the included studies are summarized in Table 1. Six observational studies were included in this meta-analysis, encompassing a total of 5,925 patients, of whom 4,841 had BMI <30 and 1,084 had BMI ≥30. The mean age across studies ranged from 50 to 78 years.

**Table 1:** Baseline Characteristics of Included Studies

**Study**	**Country**	**Study Design**	**Time frame**	**Sample Size BMI ≥30/ BMI <30, n**	**Age BMI ≥30/ BMI <30, mean or median**	**Diabetes BMI ≥30/ BMI <30, n(%)**	**Hypertension BMI ≥30/ BMI <30, n(%)**	**NYHA class III / IV BMI ≥30/ BMI <30, n(%)**
Ali-Hasan 2024	Germany	Observational	2011 - 2023	196/ 738	66.4/ 64.5†	18 (9.2)/ 18 (2.4)	NR	NR
Aljanadi 2021	United Kingdom	Observational	2011 - 2018	41/ 255	60/ 63*	1 (2.4)/ 9 (3.5)	17 (41.4)/ 79 (31)	10 (24.4)/ 73 (28.6)
Barbero 2026	Italy	Observational	2006 - 2026	149/1546	64/ 64*	42 (28.2)/ 119 (7.7)	110 (73.8)/ 852 (55.1)	97 (65.1)/ 724 (46.8)
Fiorentino 2025	Switzerland	Observational	2010 - 2024	170/ 1603	67/ 65*	32 (18.8)/ 108 (6.7)	119 (70)/ 851 (53.1)	54 (31.8)/ 412 (25.7)
Mazur 2026	Germany	Observational	1999 - 2022	501/ 501	60/ 62*	79 (15.8)/ 73 (14.6)	NR	412 (82.2)/ 408 (81.4)
Reser 2013	Switzerland	Observational	2009 - 2011	27/ 198	62/ 60.5†	6 (22.2)/ 7 (3.5)	23 (85.2)/ 99 (50)	9 (33.3)/ 53 (26.8)
*Median; † Mean; BMI: Body Mass Index; NYHA: New York Heart Association								

**Primary outcome**

**Fig. 3:**
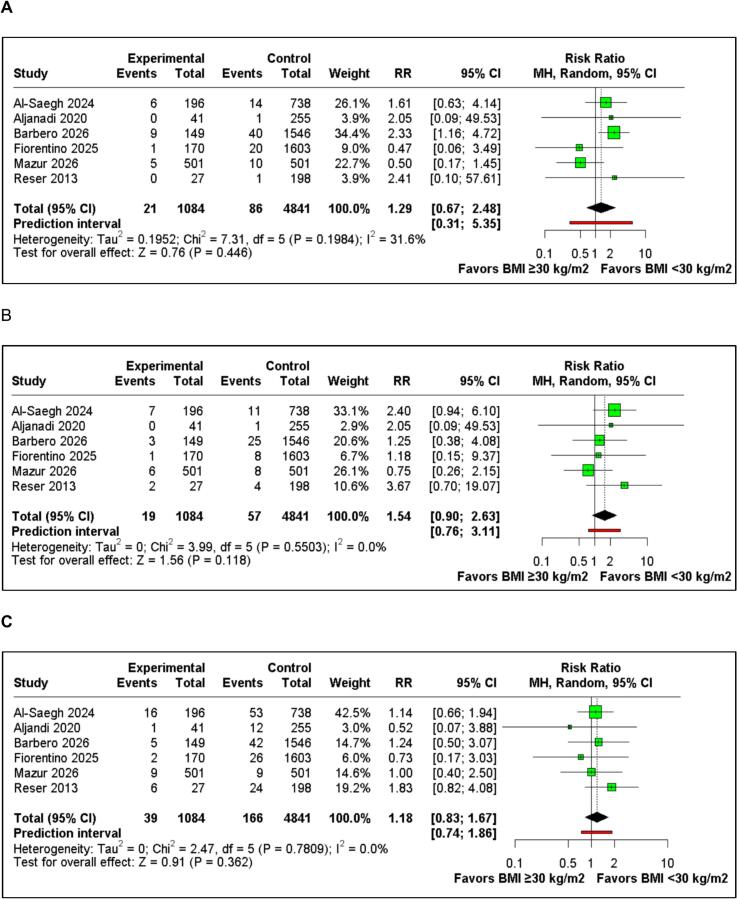
Forest plots of perioperative outcomes comparing BMI ≥30 and BMI <30 in patients undergoing MIMVS. A) In-hospital mortality. B) Stroke. C) Re-exploration for bleeding.

Table 2 summarizes the main findings of this meta-analysis. In-hospital mortality was reported in six studies including 5925 patients. The pooled analysis did not demonstrate a statistically significant difference between BMI ≥30 and BMI <30 groups (RR 1.29; 95%CI 0.67 to 2.48; p=0.446; I^2^=31.6%, Fig. 3A). The certainty of evidence for this outcome was assessed as low, downgraded for risk of bias and imprecision.

**Table 2:** Summary of outcomes.

**Outcome**	**Number of Studies**	**Number of Patients**	**Effect Estimate, Random Model (95% CI, p-value)**	**GRADE Certainty**
In-hospital mortality	6	5925	(RR 1.29; 95% CI 0.67–2.48; p=0.446; I^2^=31.6%)	⊕⊕◯◯ Low
Stroke	6	5925	(RR 1.54; 95% CI 0.90–2.63; p=0.118; I^2^=0%)	⊕⊕◯◯ Low
Re–exploration for bleeding	6	5925	(RR 1.18; 95% CI 0.83–1.67; p=0.362; I^2^=0%)	⊕⊕◯◯ Low
Duration of intubation	4	4698	(MD 0.88 hours; 95% CI 0.11–1.65; p=0.02; I^2^=35.7%)	⊕⊕◯◯ Low
ICU LOS	4	3989	(MD 0.27 days; 95% CI −0.09 to 0.62; p=0.14; I^2^=85.7%)	⊕◯◯◯ Very Low
Hospital LOS	5	4991	(MD 0.51 days; 95% CI 0.04–0.99; p=0.03; I^2^=82.3%)	⊕◯◯◯ Very Low
Postoperative atrial fibrillation	6	5925	(RR 1.28; 95% CI 1.12–1.46; p<0.001; I^2^=0%)	⊕⊕◯◯ Low
Permanent pacemaker implantation	6	5925	(RR 1.43; 95% CI 1.01–2.01; p=0.043; I^2^=0%)	⊕⊕◯◯ Low
Wound complications	6	5925	(RR 1.95; 95% CI 1.00–3.78; p=0.050; I^2^=68.6%)	⊕◯◯◯ Very Low
CI: confidence interval, MD: Mean difference, LOS: Length of stay

**Secondary outcomes**

Six studies, encompassing 5,925 patients, reported stroke. The pooled analysis did not demonstrate a statistically significant difference between the BMI ≥30 and BMI <30 groups (RR 1.54; 95%CI 0.90 to 2.63; p=0.118; I^2^=0%; Fig. 3B).

Six studies, encompassing 5,925 patients, reported re-exploration for bleeding. The pooled analysis did not demonstrate a statistically significant difference between the BMI ≥30 and BMI <30 groups (RR 1.18; 95%CI 0.83 to 1.67; p=0.362; I^2^=0%; Fig. 3C).

**Fig. 4:**
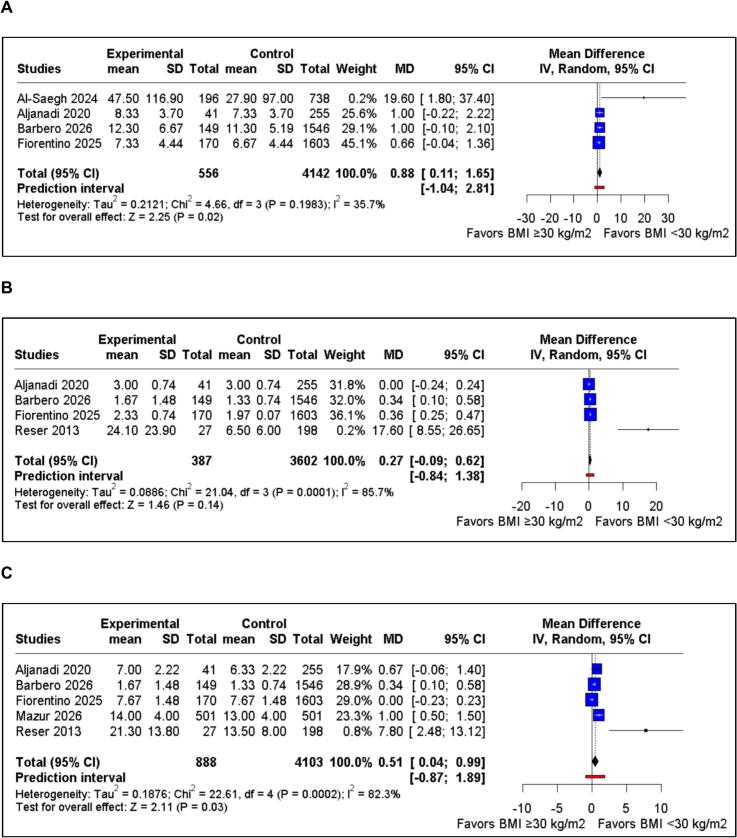
Forest plots of postoperative recovery outcomes comparing BMI ≥30 and BMI <30 in patients undergoing MIMVS. A) Duration of intubation. B) Intensive care unit length of stay. C) Hospital length of stay.

Four studies, encompassing 4,698 patients, reported duration of intubation. The pooled analysis demonstrated a significantly longer duration of intubation in the BMI ≥30 group compared with the BMI <30 group (MD 0.88 hours; 95%CI 0.11 to 1.65; p=0.02; I^2^=35.7%; Fig. 4A).

Four studies, encompassing 3,989 patients, reported ICU LOS. The pooled analysis did not demonstrate a statistically significant difference between the BMI ≥30 and BMI <30 groups (MD 0.27 days; 95%CI −0.09 to 0.62; p=0.14; I^2^=85.7%; Fig. 4B).

Five studies, encompassing 4,991 patients, reported hospital LOS. The pooled analysis demonstrated a significantly longer hospital LOS in the BMI ≥30 group compared with the BMI <30 group (MD 0.51 days; 95%CI 0.04 to 0.99; p=0.03; I^2^=82.3%; Fig. 4C).

**Fig. 5:**
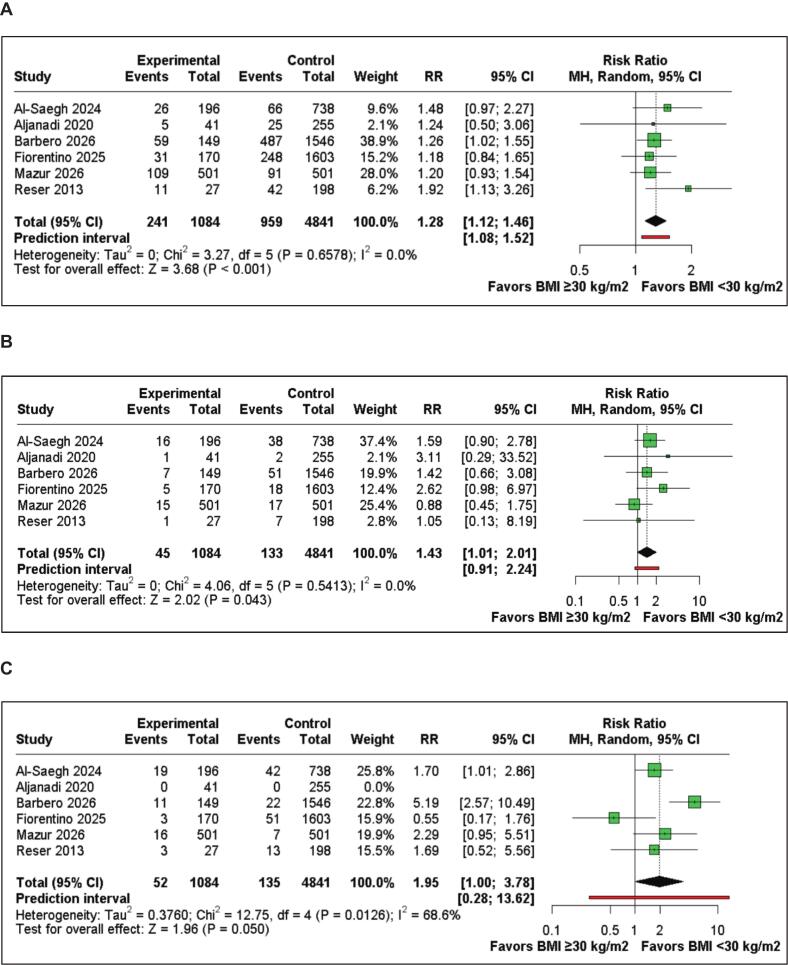
Forest plots of perioperative complications comparing BMI ≥30 and BMI <30 in patients undergoing MIMVS. A) Postoperative atrial fibrillation. B) Permanent pacemaker implantation. C) Wound complications.

Six studies, encompassing 5,925 patients, reported POAF. The pooled analysis demonstrated a significantly higher incidence in the BMI ≥30 group compared with the BMI <30 group (RR 1.28; 95%CI 1.12 to 1.46; p<0.001; I^2^=0%; Fig. 5A).

Six studies, encompassing 5,925 patients, evaluated the requirement for PPI. The pooled analysis demonstrated a significantly higher requirement for PPI in the BMI ≥30 group (RR 1.43; 95% CI 1.01–2.01; p=0.043; I^2^=0%; Fig. 5B).

Six studies, encompassing 5,295 patients, reported wound complications. The pooled analysis did not demonstrate a statistically significant difference between the BMI ≥30 and BMI <30 groups (RR 1.95; 95%CI 1.00 to 3.78; p=0.050; I^2^=68.6%; Fig. 5C).

## 5 Sensitivity Analyses

Leave-one-out analyses were performed for outcomes with significant heterogeneity to assess the influence of individual studies on the pooled estimates. Overall, the direction and magnitude of the effects remained stable, and no single study substantially altered the main conclusions. For ICU LOS (Supplementary Fig. 4), hospital LOS (Supplementary Fig. 5), pooled mean differences remained consistent. For wound complications (Supplementary Fig. 6), pooled risk ratios also remained consistent after exclusion of individual studies. Overall, these analyses confirmed the robustness of the findings, with no individual study exerting undue influence on the pooled estimates.

## 6 DISCUSSION

In this systematic review and meta-analysis of 5,925 patients undergoing MIMVS, obesity (BMI ≥30 kg/m^2^) was not associated with a statistically significant increase in in-hospital mortality compared with BMI <30 kg/m^2^. However, obese patients demonstrated significantly higher rates of POAF, prolonged ventilation duration, longer hospital LOS, and increased requirement for PPI. Conversely, no significant differences were observed in stroke, ICU LOS, re-exploration for bleeding, or wound complications.

These findings are consistent with the broader literature evaluating obesity in cardiac surgery and MIMVS. Mazur et al. [14], in a large propensity-matched cohort from a high-volume German center, demonstrated that obesity did not adversely affect repair durability or long-term survival following minimally invasive mitral repair. Similarly, Barbero et al. [15] reported comparable perioperative mortality between obese and non-obese patients across a 20-year institutional experience, while Aljanadi et al. [7] also observed no significant mortality difference in a smaller single-center cohort. In contrast, Ali-Hasan et al. [1] identified obesity as an independent predictor of postoperative complications, including respiratory insufficiency and arrhythmias, findings that parallel the increased rates of POAF and prolonged ventilation observed in our pooled analysis.

The absence of increased mortality among obese patients undergoing MIMVS may partially reflect the well-described “obesity paradox” observed in cardiovascular surgery. Several mechanisms may explain this phenomenon. First, BMI is an imperfect surrogate for adiposity and does not distinguish adipose tissue from lean muscle mass; patients with greater metabolic and nutritional reserve may better tolerate the physiological stress associated with cardiac surgery [16]. Second, obese patients may become symptomatic earlier during mitral valve disease, potentially resulting in referral for intervention at a less advanced hemodynamic stage and with greater physiological reserve [3], [16]. Third, lower BMI categories in cardiac surgery cohorts frequently include patients with occult frailty, sarcopenia, cachexia, or chronic systemic illness, which may contribute to worse postoperative outcomes independent of surgical risk [16]. Collectively, these factors suggest that elevated BMI alone should not be interpreted as a marker of prohibitive perioperative risk in the context of MIMVS.

From a technical perspective, MIMVS in obese patients remains inherently more demanding than in non-obese individuals. Increased chest wall thickness augments the distance between the right mini-thoracotomy incision and the mitral valve, potentially limiting instrument reach and maneuverability through minimally invasive ports [8]. In addition, abundant subcutaneous and mediastinal adiposity may compromise exposure, while obesity may complicate femoral artery cannulation for peripheral cardiopulmonary bypass [8]. Impaired respiratory mechanics and reduced pulmonary compliance further increase the complexity of single-lung ventilation and perioperative anesthetic management [8]. Despite these anatomical and technical challenges, our pooled analysis did not demonstrate higher rates of wound complications, ICU LOS, or re-exploration for bleeding in obese patients, supporting the feasibility and safety of MIMVS in experienced centers using standardized perioperative pathways.

The increased incidence of POAF observed in obese patients is biologically plausible and consistent with prior literature linking obesity to atrial remodeling, autonomic dysfunction, systemic inflammation, and left atrial enlargement [17], [19], [20], [21]. Similarly, the higher requirement for PPI may may suggest an association between obesity and increased conduction-related vulnerability mediated by metabolic dysregulation, adiposity-related myocardial infiltration, chronic inflammatory activation, or increased technical complexity during minimally invasive valve procedures [18], [20]. These findings suggest that obesity may disproportionately influence electrophysiological and rhythm-related outcomes rather than global postoperative recovery.

Although stroke was not significantly increased in the pooled analysis, the point estimate suggested a numerically higher risk among obese patients, which remains clinically relevant. Obesity is strongly associated with endothelial dysfunction, platelet activation, hypercoagulability, and systemic inflammation, all of which contribute to cerebrovascular risk [3], [16]. The absence of statistical significance may therefore reflect insufficient power rather than the absence of a true association, particularly given the relatively low event rates across included studies.

The significantly longer duration of mechanical ventilation and hospital LOS in obese patients likely reflects the cumulative impact of impaired pulmonary mechanics, reduced chest wall compliance, higher prevalence of obstructive sleep apnea, and greater perioperative respiratory management complexity. Nevertheless, the absolute differences were modest, and no significant increase in ICU LOS was observed, suggesting that obesity may prolong recovery without substantially increasing severe postoperative morbidity when contemporary perioperative care pathways are applied.

The substantial heterogeneity observed for ICU LOS, hospital LOS, and wound complications likely reflects differences in institutional discharge practices, enhanced recovery protocols, patient selection, and surgical expertise across centers and study periods. Importantly, sensitivity analyses confirmed the robustness of the pooled estimates, with no individual study exerting disproportionate influence on the overall findings.

Overall, these findings support a nuanced interpretation of obesity in MIMVS. While obesity appears associated with higher rates of rhythm-related complications and modestly prolonged postoperative recovery, it was not associated with increased in-hospital mortality, wound complications, or major perioperative morbidity. Taken together, these results suggest that obesity alone should not be considered a contraindication to MIMVS, particularly when procedures are performed in experienced centers with structured perioperative management and careful postoperative monitoring for arrhythmic and respiratory complications.

## 7 Limitations

This meta-analysis has several limitations. All included studies were observational, introducing the potential for residual confounding and selection bias despite adjustment for baseline characteristics. BMI was used as the primary measure of adiposity, although it does not account for body composition, visceral fat distribution, or sarcopenia, which may more accurately reflect cardiometabolic risk. Considerable heterogeneity was observed for several continuous outcomes, likely reflecting variations in perioperative pathways, discharge criteria, and institutional expertise across centers performing MIMVS. In addition, low event rates for outcomes such as stroke, wound complications, and PPI resulted in relatively wide confidence intervals, limiting precision. The included studies spanned more than two decades, during which MIMVS techniques, perfusion strategies, and enhanced recovery protocols evolved substantially, potentially affecting generalizability. Furthermore, only English-language studies were included, which may have introduced language bias and led to exclusion of potentially relevant non-English evidence. Finally, limited granularity regarding operative complexity, cannulation strategy, myocardial protection techniques, and comorbidity burden restricted the ability to perform more detailed subgroup analyses exploring technical determinants of perioperative outcomes.

## 8 CONCLUSION

In patients undergoing MIMVS, obesity did not appear to be associated with increased in-hospital mortality but was associated with higher rates of POAF and modestly prolonged postoperative recovery. These findings suggest that obesity alone should not preclude MIMVS, although careful perioperative monitoring remains warranted.

DECLARATIONS

## CRediT authorship contribution statement

**Paul C. Onyeji:** Writing – original draft, Methodology, Investigation, Data curation, Conceptualization. **Sonise Momplaisir-Onyeji:** Writing – original draft, Validation, Formal analysis, Data curation. **Abhigyan Majumdar:** Software, Methodology, Investigation. **Przemysław Nowakowski:** Visualization, Validation, Software. **Felipe S. Passos:** Writing – review & editing, Supervision, Methodology. **Hristo Kirov:** Writing – review & editing, Supervision. **Ricardo Esper Treml:** Writing – review & editing, Validation. **Torsten Doenst:** Writing – review & editing. **Sophie Tkebuchava:** Writing – review & editing, Validation. **Tulio Caldonazo:** Writing – review & editing, Visualization, Validation, Funding acquisition.

## Funding

TC was funded by the Deutsche Forschungsgemeinschaft (DFG, German Research Foundation) Clinician Scientist Program OrganAge funding number 413668513, by the Deutsche Herzstiftung (DHS, German Heart Foundation) funding number S/03/23 and by the Interdisciplinary Center of Clinical Research of the Medical Faculty Jena.

## Declaration of competing interest

The authors declare that they have no known competing financial interests or personal relationships that could have appeared to influence the work reported in this paper.

## Appendix A Supplementary data

Supplementary Data 1 The supplementary material provides additional methodological details and sensitivity analyses supporting the main findings. It includes the complete search strategy used across databases (Supplementary Table 1) and leave-one-out sensitivity analyses for ICU length of stay, hospital length of stay, and wound complications (Supplementary Figures 1–3).
