# Supplementary material for: Impact of Obesity on Outcomes after Minimally Invasive Mitral Valve Surgery: A Systematic Review and Meta-Analysis
Source: Int J Cardiol Heart Vasc. 2026 May 22;64:101944. doi: 10.1016/j.ijcha.2026.101944 (PMC13224114; doi:10.1016/j.ijcha.2026.101944)

**Supplementary material**

Supplementary Table 1 – Complete search strategy

Supplementary Figure 1 – Leave-one-out analysis for ICU LOS

Supplementary Figure 2 – Leave-one-out analysis for hospital LOS

Supplementary Figure 3 – Leave-one-out for wound complications

**Supplementary Table 1 – Complete search strategy**

Search: ("mitral valve surgery" OR "mitral valve repair" OR "mitral valve replacement") AND ("minimally invasive" OR "mini-thoracotomy" OR "robotic" OR "video-assisted") AND ("obesity" OR "body mass index" OR BMI OR obese)

("mitral valve surgery"[All Fields] OR "mitral valve repair"[All Fields] OR "mitral valve replacement"[All Fields]) AND ("minimally invasive"[All Fields] OR "mini-thoracotomy"[All Fields] OR "robotic"[All Fields] OR "video-assisted"[All Fields]) AND ("obesity"[All Fields] OR "body mass index"[All Fields] OR "BMI"[All Fields] OR ("obeses"[All Fields] OR "obesity"[MeSH Terms] OR "obesity"[All Fields] OR "obese"[All Fields] OR "obesities"[All Fields] OR "obesity s"[All Fields]))

Translations

obese: "obeses"[All Fields] OR "obesity"[MeSH Terms] OR "obesity"[All Fields] OR "obese"[All Fields] OR "obesities"[All Fields] OR "obesity's"[All Fields]

Supplementary Figure 1 – Leave-one-out analysis for ICU length of stay


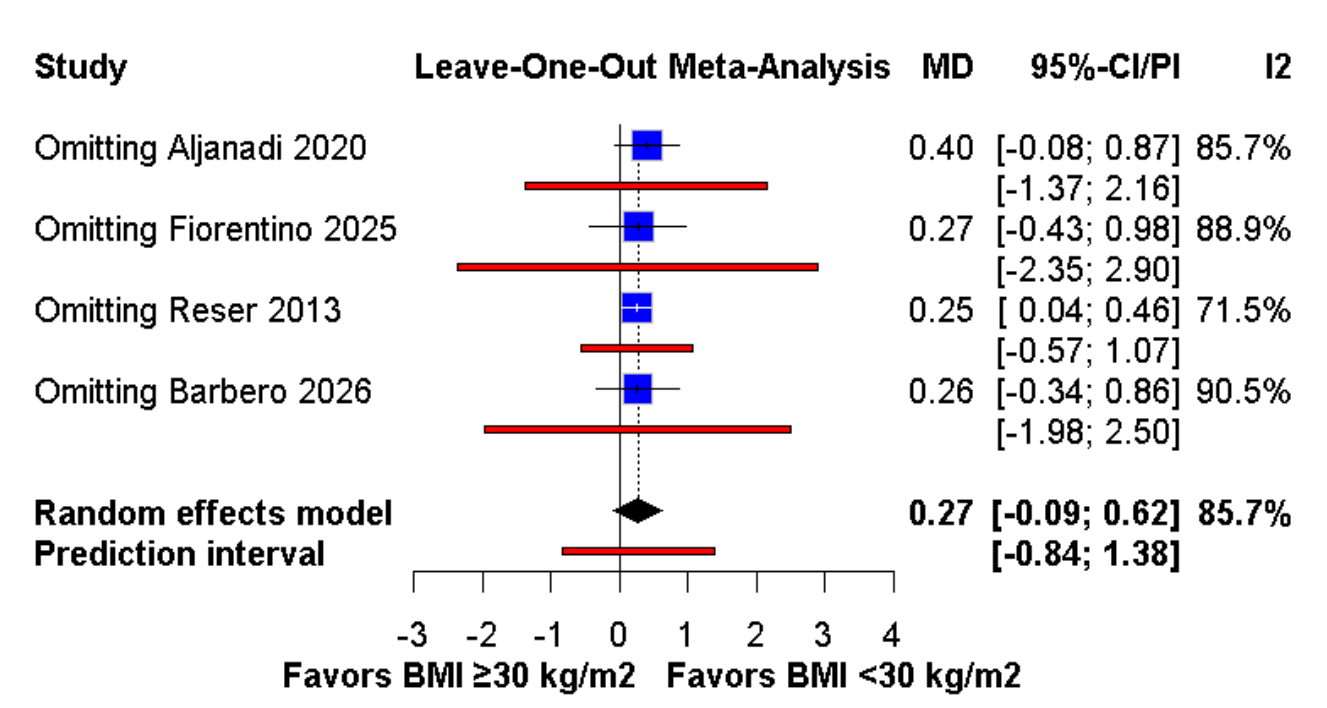


Supplementary Figure 2– Leave-one-out analysis for hospital LOS


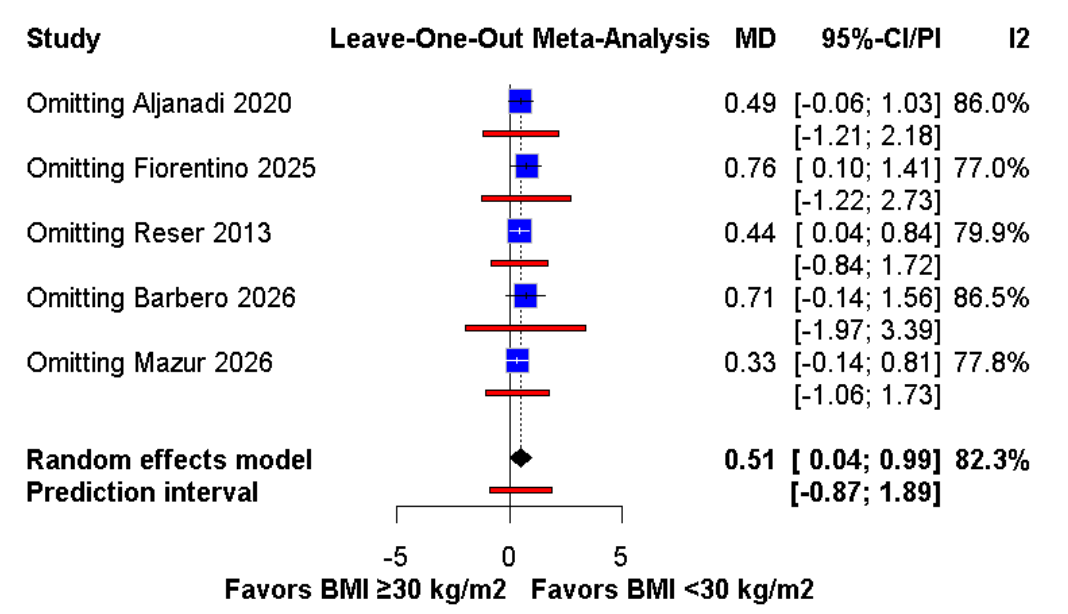


Supplementary Figure 3 – Leave-one-out for wound complications


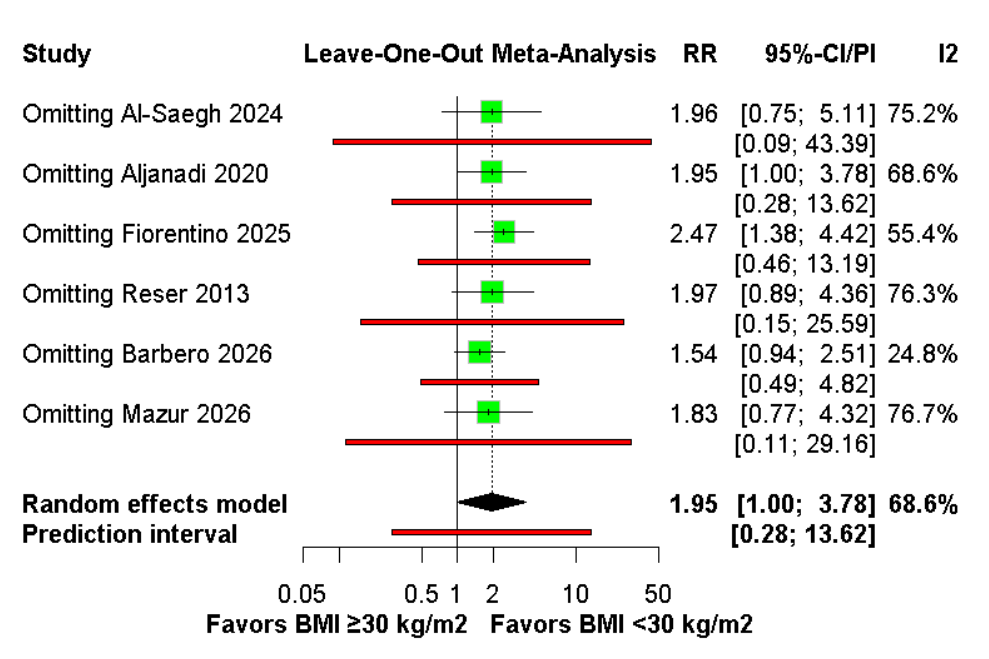

Supplement: Supplementary Data 1 — The supplementary material provides additional methodological details and sensitivity analyses supporting the main findings. It includes the complete search strategy used across databases (Supplementary Table 1) and leave-one-out sensitivity analyses for ICU length of stay, hospital length of stay, and wound complications (Supplementary Figures 1–3). [file mmc1.docx]
